# Mechatronic Development and Vision Feedback Control of a Nanorobotics Manipulation System inside SEM for Nanodevice Assembly

**DOI:** 10.3390/s16091479

**Published:** 2016-09-14

**Authors:** Zhan Yang, Yaqiong Wang, Bin Yang, Guanghui Li, Tao Chen, Masahiro Nakajima, Lining Sun, Toshio Fukuda

**Affiliations:** 1 Jiangsu Key Laboratory for Advanced Robotics, Soochow University, Suzhou 215123, China; yangzhan@suda.edu.cn (Z.Y.); 20154229003@stu.suda.edu.cn (Y.W.); 15151541352@163.com (G.L.); lnsun@hit.edu.cn (L.S.); 2 Collaborative Innovation Center of Suzhou Nano Science and Technology, Soochow University, Suzhou 215123, China; 3 School of Mechatronic Engineering and Automation, Shanghai University, Shanghai 200072, China; yangbinfree@163.com; 4 Department of Micro-Nano Systems Engineering, Nagoya University, Nagoya 464-8603, Japan; nakajima@mein.nagoya-u.ac.jp (M.N.); fukuda@mein.nagoya-u.ac.jp (T.F.); 5 Intelligent Robotics Institute, School of Mechatronic Engineering, Beijing Institute of Technology, Beijing 100081, China

**Keywords:** carbon nanotube, nanorobotics manipulation, mechatronics design and development, visual feedback

## Abstract

Carbon nanotubes (CNT) have been developed in recent decades for nanodevices such as nanoradios, nanogenerators, carbon nanotube field effect transistors (CNTFETs) and so on, indicating that the application of CNTs for nanoscale electronics may play a key role in the development of nanotechnology. Nanorobotics manipulation systems are a promising method for nanodevice construction and assembly. For the purpose of constructing three-dimensional CNTFETs, a nanorobotics manipulation system with 16 DOFs was developed for nanomanipulation of nanometer-scale objects inside the specimen chamber of a scanning electron microscope (SEM). Nanorobotics manipulators are assembled into four units with four DOFs (X-Y-Z-θ) individually. The rotational one is actuated by a picomotor. That means a manipulator has four DOFs including three linear motions in the X, Y, Z directions and a 360-degree rotational one (X-Y-Z-θ stage, θ is along the direction rotating with X or Y axis). Manipulators are actuated by picomotors with better than 30 nm linear resolution and <1 micro-rad rotary resolution. Four vertically installed AFM cantilevers (the axis of the cantilever tip is vertical to the axis of electronic beam of SEM) served as the end-effectors to facilitate the real-time observation of the operations. A series of kinematic derivations of these four manipulators based on the Denavit-Hartenberg (D-H) notation were established. The common working space of the end-effectors is 2.78 mm by 4.39 mm by 6 mm. The manipulation strategy and vision feedback control for multi-manipulators operating inside the SEM chamber were been discussed. Finally, application of the designed nanorobotics manipulation system by successfully testing of the pickup-and-place manipulation of an individual CNT onto four probes was described. The experimental results have shown that carbon nanotubes can be successfully picked up with this nanorobotics manipulation system.

## 1. Introduction

Since the discovery of carbon nanotubes (CNTs) in 1991 [1], their well-defined geometries, mechanical and electrical properties have been extensively investigated. The properties of carbon nanotubes have been briefly summarized in [2]. Those exceptional properties suggest broad potential application for carbon nanotubes. They can be used in the bulk state to synthesize conductive and high-strength composites [3], to fabricate field emission devices [4], to save and covert electrochemical energy [5], and so on. However, the most promising application of nanotubes is to maneuver the tubes individually to build complex nanodevices including nanoelectronics and nanoelectromechanical systems (NEMS). Examples of such devices are varied. Carbon nanotube as a probe for friction force microscopy was demonstrated by Ishikawa et al. [6]. Gas sensors based on changes in the dielectric constant rather than the electrical conductivity of single walled carbon nanotubes (SWNTs) have also been demonstrated [7]. For nanoelectronics, carbon nanotube field-effect transistors (CNTFETs) have been promoted as a replacement for silicon technology. This first demonstration of CNT transistors with channel lengths down to 9 nm has shown substantially better scaling behavior than theoretically expected [8].

The extreme small-scale and variety of CNTs provide certain challenges for the development of new tools for fabricating them, manipulation methods and properties measurements. In order to build nanodevices with CNTs, along with other nanoscale components, such objects must presently be examined, characterized, and controllably manipulated into position within an integrated device. The potential of engineering application of CNTs to fabricate nanometer-scale electronic devices and mechanical systems largely depends on the capability of manipulating them in three-dimensional space. Thus, nanorobotics manipulation [9] is proved to be the most promising way for nanodevice construction and assembly. Although robotic manipulation and control strategies are successfully applied in macro-scale applications [10,11], a major nanotechnology obstacle faced today is the lack of effective processes for building the nanoscale structures needed for the envisaged applications. Researchers have shown strategies and systems for 3D nanorobotics manipulation of carbon nanotubes, and the nanoassembly of multi-walled carbon nanotubes (MWNTs) in [2]. Guthold et al. established a nanomanipulation system based on an atomic force microscope (AFM) to investigate nanotubes, DNA, adeno- and tobacco-mosaic virus. In their work, nanotubes have been bent, translated and rotated to show their mechanical properties [12]. Shen designed a robot with endless rotation ability for material characterization [13]. In manipulation via a nanorobotics manipulator, much more DOFs, including rotation, for orientation control of the nanoparticle are feasible. For this reason, robotic manipulators can be used for manipulations in 3D space and much progress has been made in nanorobotics manipulation recently [14] Dong et al. realized 3D nanomanipulations of nano-order objects with a 10 DOFs nanorobotics manipulator, which is actuated by PZTs and picomotors and operated inside a scanning electron microscope (SEM). 3D manipulations of multiwall carbon nanotubes were performed, including picking up and bending singles ones [15]. Fukuda et al. accomplished assembly of nanodevices with carbon nanotubes through nanorobotics manipulations inside a SEM. A nanorobotics manipulator with 16 DOFs has been designed for complex manipulation [2]. The assembly of nanodevices with multi-walled carbon nanotubes was presented. Manipulations such as picking up carbon nanotubes, in situ property characterization, destructive fabrication and shape modification were implemented under the real-time observation inside a SEM [16]. Saeipourazar and Jalili designed a fused vision force feedback control for a nanorobotics-based manipulator for nanofiber grasping [17] and nano-fabric production applications [18]. The systems mentioned above were teleoperated manipulation systems, which were able to transform the user′s hand motion into the finer 3D motions of the system manipulators by a sophisticated man-machine user interface. With the development of the technology, systems with automated nanohandling desktop stations arose for high-efficiency nanomanipulation. Fatikow et al. set up a nanorobotics system that could be used to realize automated handling and characterization as well as prototyping of CNT-based components [19]. Shen proposed a method that can fill the holes and noises of the depth map to keep the edges smooth based on an integrated nanorobotics AFM and SEM system [20]. Sun et al. conducted automated nanomanipulation inside an SEM for a well-structured nanomanipulation task via visual servo-control and achieved four-point probe measurement of individual nanowires automatically by controlling four nanomanipulators with SEM visual feedback [21]. Zhou et al. provided automated probing of nanostructures inside an SEM with a probing accuracy better than 3 nm and a drift rate lower than 1 nm/min [22]. The Xie group presented high-efficiency automated nanomanipulation with a parallel imaging/manipulation force microscope (PIMM) [23]. A vision-based nanorobotics system has also been developed and integrated by Shen et al. [24].

Since nanotubes have great electrical conductivity which is 1000 times greater than that of copper, they are the perfect candidates for novel interconnects [25] and nanoelectronic applications [26]. CNTFETs have been experimentally shown to provide superb low-voltage performance with channel lengths scaled into the sub-10 nm regime [27]. Because of the size effects, the structure of field effect transistors (FETs) can be expanded from two to three dimensions. Three-dimensional FETs will be component units of future electronic devices. Moreover, the novel three-dimensional FETs based on CNTs may offer extraordinary properties in electronic devices. Our ultimate goal is to construct three-dimensional CNTFETs for computing technology. Hence, a nanorobotics manipulation system should be designed and developed for constructing nanodevices. Generally speaking, high-efficiency nanomanipulation requires multiplr degrees of freedom, including rotation, for orientation control of nanotubes and automated nanohandling control. Besides, a single nanomanipulator itself has limited capabilities, but the coordinated effort of a multitude will produce the desired system-level results. Coordination is needed across the board—for communication, sensing, and acting—which poses a major research challenge. The scale and dynamics of nanorobotics systems precludes centralized coordination and global sharing of states. Therefore, we need coordination schemes that are inherently distributed and based on localized inputs, algorithms, and outputs.

In this paper, a nanorobotics manipulation system providing the possibilities of pickup-and-place manipulation and automation handling sequences by integrating specially developed techniques and strategies was described. It was integrated into an SEM and could use both the tip of the AFM probe for the manipulation of CNTs as well as other operation end-effectors for CNT handling. The designed nanorobotics manipulation system has 16 DOFs for 3D nanomanipulation of nanometer-scale objects and the construction of complex nanodevices. In Section 1, we introduce some previous research on nanomanipulation systems. The detailed structure of the nanorobotics manipulation system is described in Section 2. Electrical connection and driving control between tools and controllers inside and outside the SEM are introduced in Section 2 as well. A theoretical model of each nanorobotics manipulator is established based on the Denavit-Hartenberg (D-H) notation in Section 3. The common working space of manipulators is also calculated. In Section 4, the whole control system architecture is described. The manipulation strategy and vision feedback control for multi-manipulators operating inside the SEM chamber are also discussed. Finally, application of the designed nanorobotics manipulation system by successfully testing of the pickup-and-place manipulation of an individual CNT onto four probes is described in Section 5. This system is partially automated up to now and proves the concept of the discussed integrated nanorobotics manmipulators and operation strategies.

## 2. System Setup

### 2.1. Mechanical Design and Developments of Nanorobotics Manipulation System

A manipulation device inside a SEM must perform a variety of functions for handling, characterizing, deforming and building with CNTs and other small components. It should possess the ability and precision to probe a collection of CNTs, confirm it is an ordered array or disordered bunch, and then isolate a CNT and extract it from the collection. Once a CNT is isolated from the group, the manipulator must permit operations, both mechanical and electrical. It′s necessary to characterize the strength and conductivity of the length of the individual CNT that is free from contact with a surface. Ideally, stretching, bending, twisting and compressing the tube at designated locations along the tube should be possible. The manipulator probes performing these operations should be metallic or metal-coated so that conductivity measurements can be carried out simultaneously. When suitable components are selected, the manipulator should permit transferring and construction operations, such as building three-dimensional CNT structures. It must further perform these operations without interfering mechanically with the microscope′s components and without disturbing the electron microscope′s imaging quality.

**Figure 1:**
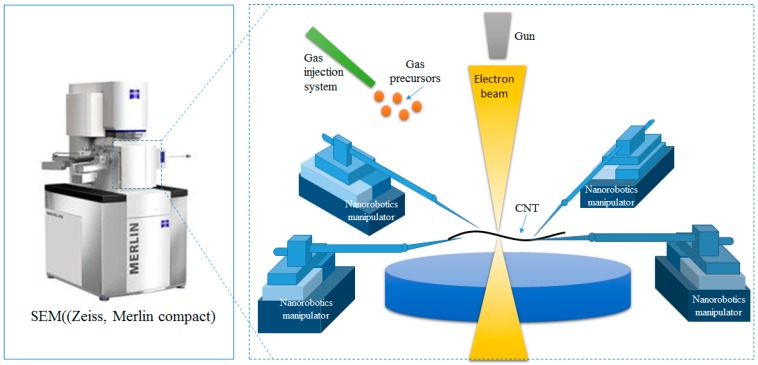
SEM based nanorobotics manipulation system.

**Figure 2:**
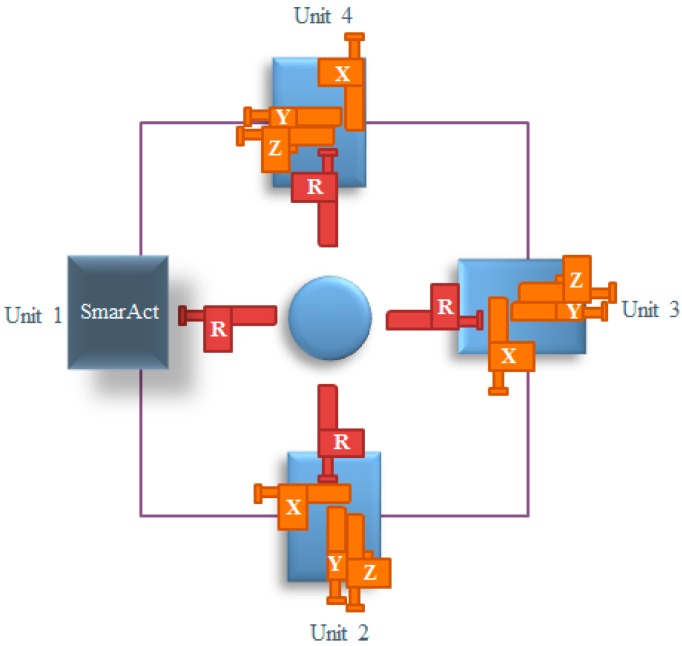
Four units of the nanorobotics manipulation system.

In order to manipulate nanoscale objects with AFM cantilever in 3D space for constructing nanostructures and devices inside an SEM, a SEM system with 16 degrees of freedom was introduced to obtain a real-time monitor for nanomanipulation. The image resolution of the SEM is about 1.5 nm with an accelerating voltage of 30 kV. Figure 1 indicates the nanorobotics manipulation system designed inside the SEM for the purpose of constructing three-dimensional CNTFETs. Four nanorobotics manipulators were symmetrically distributed and pairwisely set opposite to each other inside the SEM. The nanorobotics manipulators were divided into four units named Unit 1, Unit 2, Unit 3, Unit 4, respectively, as depicted in Figure 2.

The designed nanorobotics manipulation system with 16 DOFs was constructed for 3D nanomanipulation of nanometer-scale objects and 3D nanodevices construction inside a specimen chamber of the SEM. A SEM (Merlin compact, resolution 0.8 nm @15 kV, Zeiss, Jena, Germany) was introduced to obtain a real-time monitor for nanomanipulation. Nanorobotics manipulators were assembled into four units. Unit 1 consists of a three-axes micromanipulator (SLC-1720-s, SmarAct, Oldenburg, Germany), a Picomotor^TM^ (8301-UHV, New Focus Inc., San Jose, CA, USA) and an atomic force microscope (AFM) cantilever (OMCL-TR400PB-1, Olympus, Tokyo, Japan). The construction of the other units is the same, including a three-dimensional (X, Y, Z) micro-motion platform (TSDS-255C, Sigma, Tokyo, Japan), four 8301-UHV Picomotors^TM^ (New Focus Inc.) and an atomic force microscope (AFM) cantilever (Olympus OMCL TR400PB-1) as an end-effector. The movements of the X, Y, Z stages in the three directions were actuated by three picomotors which could turn the rotary motion of picomotors into linear motion. The rotational one was also actuated by a picomotor. That means each unit has a 4-DOF with three linear motions in the X, Y, Z directions and a 360-degree rotational one (X-Y-Z-θ stage, θ is along the direction rotating with X or Y axis). The constructed three-dimensional nanorobotics manipulators are actuated with picomotors with better than 30 nm linear resolution and <1 micro-rad rotary resolution. Four AFM cantilevers serve as the end-effectors which were vertically installed (the axis of the cantilever tip is vertical to the axis of electronic beam of SEM) to facilitate the real-time observation of operation. The main parameters of the nanorobotics manipulators are listed in Table 1. These four units were driven by different actuators and capable of working independently. The sample stage was set in the center of the four units. Usually, the sample such as carbon nanotubes can be placed on the sample stage, and then the position of each end effector can be easily adjusted to satisfy the manipulation on the object.

### 2.2. Electrical Connections and Driving

**Figure 3:**
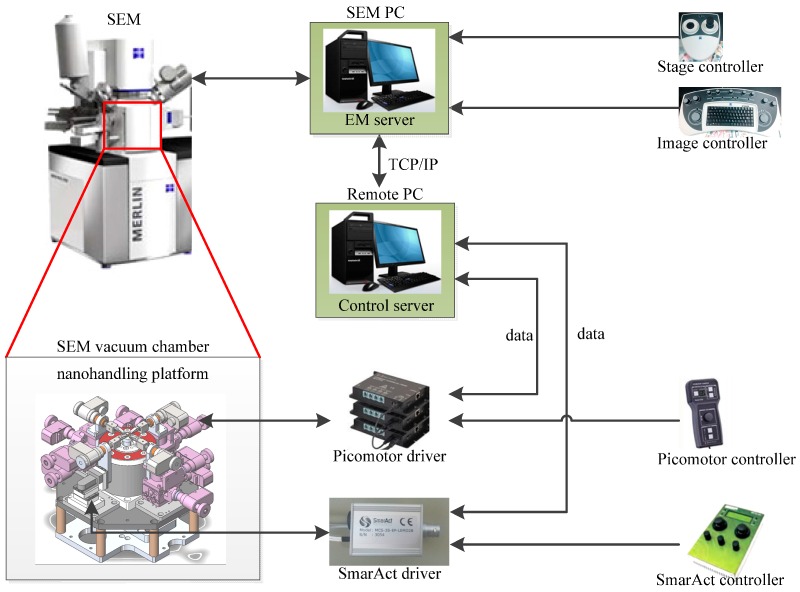
Architecture of the control system.

The space of the vacuum chamber inside the scanning electron microscopy is large enough for three-dimensional nanorobotics manipulator operation. To realize nanorobotics manipulators operating in the vacuum chamber of an SEM, some environmental challenges need to be solved. The SEM works under a high vacuum state, which means all tools and objects have to be vacuum compliant. Nanorobotics manipulators can be operated under the same vacuum conditions as the SEM. It takes several minutes to generate a high vacuum which is a serious time constraint. Therefore, the four nanorobotics manipulators and objects need to be installed inside the SEM before operation starts. In addition, the motors of the manipulators are driven by controllers outside the SEM vacuum chamber since the vacuum chamber space is limited. Thus, an electrical connection between the motors inside the vacuum chamber of the SEM and the controllers outside is needed. Figure 3 shows the electric connections for driving the motors and performing tests.

To effectively realize teleoperation of the manipulators inside the SEM, a circuit switching device was constructed. The hatch of the vacuum chamber was redesigned to meet the requirements for circuit transfer. In this case, two identical aviation plugs (MX33, Beijing Longines Electrical Equipment Co., Beijing, China) core were installed on the door of the vacuum chamber. The control lines of the motors needed to be drawn from the SEM chamber, then transferred one-to-one by the electric connection device. As a result, the controllers outside the SEM can drive the motors inside the SEM.

The nanorobotics manipulation control system is indicated in Figure 3. Treating the SEM computer as the server-side, and the remote computer as the client-side, we use TCP/IP communication for remote control. EM Server is a core service of the controlled scanning electron microscope, which includes all parameters and control information for scanning electron microscopy. All parameters such as the pixel size, brightness, contrast and magnification can be accessed directly permitting the EMServer to give commands to control the scanning electron microscope. Control Server is the core of the entire remote control system. It can obtain the electron microscope images and position information of actuators, and issue control commands to control the the scanning electron microscope and the actuator, realizing the whole closed-loop control system.

The scanning electron microscope image was obtained by Vision data from the EM Server through TCP/IP communication. By means of the image processing of the scanning electron microscope images, the positions of the CNT and AFM end-effector can be detected and obtained. Position data gets the location information of all picomotors and actuators in real time feedback.

## 3. Theoretical Model

### 3.1. Theoretical Model of the Nanorobotics Manipulator

**Figure 4:**
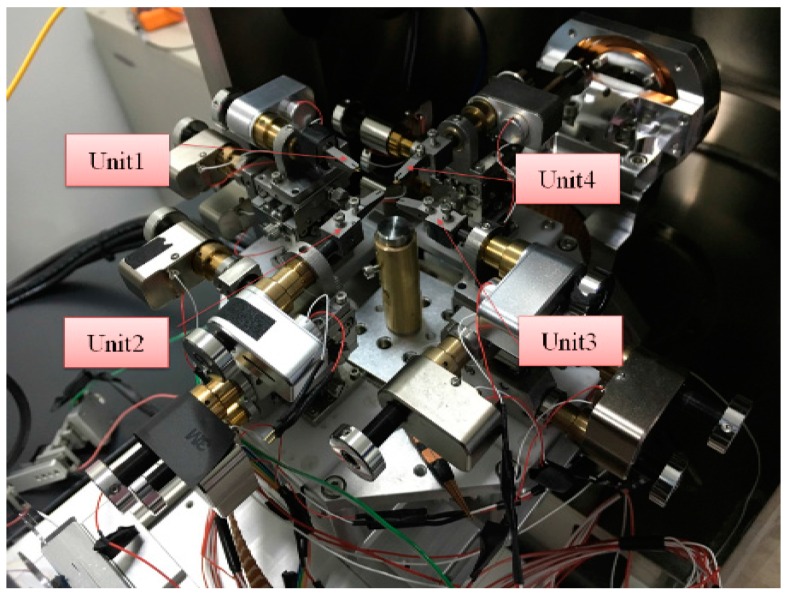
The designed nanorobotics manipulators inside the SEM.

Since these four units have a similar structure despite of their different positions, here we just introduce the design and kinematics of nanomanipulator Unit 1 as a representative example. Figure 4 displays the real structure of the designed nanorobotics manipulators inside the SEM. Unit 1 with a three-axes micromanipulator (SmarAct, SLC-1720-s) and a Picomotors^TM^ (New Focus Inc., 8301-UHV) has four DOFs with three linear motions in the X, Y, Z directions and a 360-degree rotational one (X-Y-Z-θ stage, θ is along the direction rotating with X axis). Unit 1 has linear strokes of 6 nm in the X, Y, Z directions respectively. The linear resolution is 30 nm (X, Y and Z stages) and that of the rotary one is <1 micro-rad. In general, robots can be regarded as a combination of a series of joints and bars, as is the nanorobotics manipulator. Each joint needs to specify a reference coordinate system, and then determine the transformation step from one joint to the next joint. All transforms from base to the first joint, then from the first joint to the second joint up to the last joint must be combined to obtain the total transform matrix of the manipulators. Denavit-Hartenberg (D-H) notation provides an effective way for getting the transformation matrix. Hence, the coordinates of endpoint can be calculated by the following formula:

(1) $$\left[\begin{array}{c} \begin{array}{c} \begin{array}{c} x_{endpoint}\\ y_{endpoint} \end{array}\\ z_{endpoint} \end{array}\\ 1 \end{array}\right]=T\left[\begin{array}{c} \begin{array}{c} x_{base}\\ y_{base}\\ z_{base} \end{array}\\ 1 \end{array}\right]$$

where *T* is the total transfrom matrix between base and the last joint.

**Figure 5:**
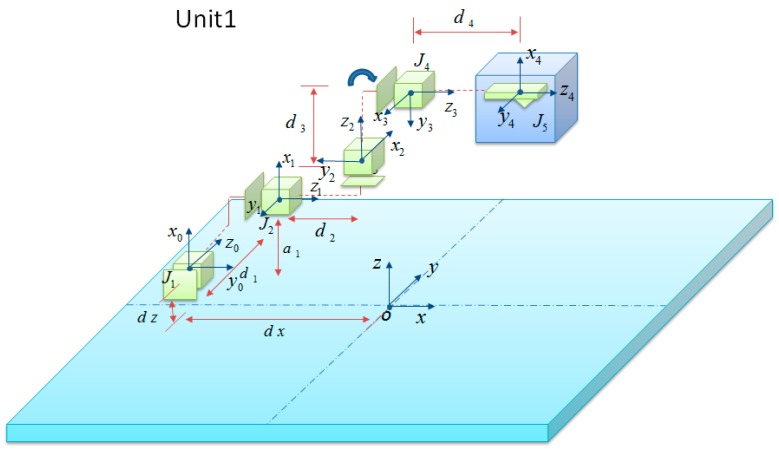
Kinematic parameters and frame assignments for the manipulator Unit 1 inside the SEM.

The following kinematics derivation of nanomanipulator Unit 1 is based on the Denavit-Hartenberg (D-H) notation. Figure 5 depicts the model of kinematics of nanomanipulator Unit 1, where $J_{i}\;\left(i=1,\;2,\;3,\;4,\;5\right)$ are joints. The nanomipulator with the AFM cantilever as an end effector was divided into five parts. The general coordinate system is established in the center of the four nanorobotics manipulators on their common base indicated with X, Y, Z frame and is coincident with the observation coordinate system. It is evident from the link frame assignments of Unit 1 in Figure 5 that Frame 1 has a translational movement in the $\left|X_{0}\right|$ axis away from frame 0. Frames 2, 3, 4 and 5 will be moved relatively to the movement of Frame 1. Frame 2 has a translational movement in the $\left|Y_{0}\right|$ axis, and its movement will drag frames 3, 4 and 5 together without affecting Frame 1. Frame 3 has a translational movement in $\left|Z_{0}\right|$ and drags frame 4 and 5 during its movement, without affecting the position or orientation of frames 1 and 2. Frame 4 can rotate around $\left|X_{0}\right|$ axis. Frame 5 is a tool frame. Link coordinate systems are depicted according to Denavit-Hartenberg (D-H) notation as shown in Figure 5. The cubic region in Figure 5 indicates the working space of the nanorobotics manipulators.

The link parameters of nanorobotics manipulator Unit 1 are listed in Table 2, where i denotes the code number of the joints, $a_{i}$ is the link length, $\alpha_{i}$ is the link twist, $d_{i}$ is the distance between links, and $\theta_{i}$ is the angle between links. The offset parameter of each frame does not list. To overcome this issue, we intentionally added the offset parameters to the transformational matrix. Because of this slight modification, we are able to include all the dimensions, including the variable and constant values, in the kinematics derivation of nanorobotics manipulator Unit 1. By defining new symbols for sin and cos as *s* and *c* respectively, we can represent the homogeneous transformations for each link as follows:

(2) T10=A1=[cθ1−sθ1cα1sα1sθ1a1cθ1sθ1cθ1cα1−sα1sθ1a1sθ10sα1cα1d10001]=[100a100100−10∆d10001]

(3) T21=A2=[cθ2−sθ2cα2sα2sθ2a2cθ2sθ2cθ2cα2−sα2sθ2a2sθ20sα2cα2d20001]=[0010−10000−10d2+∆d20001]

(4) T32=A3=[cθ3−sθ3cα3sα3sθ3a3cθ3sθ3cθ3cα3−sα3sθ3a3sθ30sα3cα3d30001]=[−100000−100−10d3+∆d30001]

(5) T43=A4=[cθ4−sθ4cα4sα4sθ4a4cθ4sθ4cθ4cα4−sα4sθ4a4sθ40sα4cα4d40001]=[sθ4cθ400−cθ4sθ400001d40001]

Finally, the homogenous transform matrix of Unit 1 is

(6) T40=T10 T21 T32 T43=A1A2A3A4=[cθ4−sθ40a1+∆d3+d3001d2+∆d2+d4−sθ4−cθ40∆d1+d10001]

where $∆d_{1}$, $∆d_{2}$, $∆d_{3}$ is the sliding transformation of each joint and $\theta_{4}$ is the rotation angle for Joint 3. The range of $\theta_{1}$ is from −360° to 360°. $a_{1}$ is the distance between J1 and J2. $d_{3}$ is the distance between J2 and J3. $d_{4}$ is the distance between J3 and J4. $a_{1}$, $d_{3}$, $d_{4}$ are constant values related to the nanorobotics manipulator’s structure. Here, $a_{1}=8.5\;\mathrm{mm}$, $d_{2}=36\;\mathrm{mm}$, $d_{3}=33.5\;\mathrm{mm}$, $d_{4}=37\;\mathrm{mm}$.

By adjusting the value of $d_{i}$ in Equation (5), the coordinate of the end-effector relative to the base coordinate system within this space can be accurate positioned. In the observation coordinate system, the coordinate of the base coordinate system is:

(7) T0U=[010−dx0010100dz0001]

where $d_{x}$ is the offset between manipulator Unit 1 and center of the four nanotobotics manipulators. Here, $d_{x}=73.5\;\mathrm{mm}$, $d_{z}=14.9\;\mathrm{mm}$.

In order to obtain a uniform coordinate transformation, the transformation between the origin point and the end point of end-effector can be indicated with T4U which was given by:

(8) T4U=T0U T40=[001d2+∆d2+d4−dx−sθ4−cθ40∆d1cθ4−sθ40a1+∆d3+d3+dz0001]

As mentioned above, four nanorobotics manipulators were exactly the same in structure except for their locations, which were symmetrically set inside the SEM. Thus, according to the calculated result about the coordinate location of the end-effector in Unit 1 relative to the origin coordinate location, the coordinates of other end-effectors mounted on Unit 2, Unit 3 and Unit 4 relative to the base coordinate system could be derived by left multiplication via a transformation matrix. The coordinates of the end-effector in Unit 2 can be deduced through rotating transformation around $\left|Z_{0}\right|$ with a rotation angle = 90°. Similarly, the coordinates of the end-effector in Unit 3 can be deduced through rotating transformation around $\left|Z_{0}\right|$ with a rotation angle = 180°. The coordinates of the end-effector in Unit 4 can be deduced through rotating transformation around $\left|Z_{0}\right|$ with a rotation angle = 270°. The transformation matrices of Unit 2, Unit 3, Unit 4 were described with T4′U, T4″U, T4‴U respectively:

(9) T4′U=Rot(z,90)T40=[−cθ4′0sθ4′−∆d2′0−10∆d1′+d4−dxsθ4′0cθ4′a1+∆d3′+d30001]

(10) T4″U=Rot(z,180)T40=[010dx−∆d1″−d4−cθ4″0sθ4″−∆d2″sθ4″0cθ4″a1+∆d3″+d30001]

(11) T4‴U=Rot(z,270)T40=[cθ4‴0−sθ4‴∆d2‴010dx−∆d1‴−d4sθ4‴0cθ4‴a1+∆d3‴+d30001]

where T4′U is the rotate angle of Unit 2. $∆d_{1}^{\prime }$, $∆d_{2}^{\prime }$, $∆d_{3}^{\prime }$ are the sliding transformations of each joint of Unit 2. T4″U is the rotate angle of Unit 3. $∆d_{1}^{\prime \prime }$, $∆d_{2}^{\prime \prime }$, $∆d_{3}^{\prime \prime }$ are the sliding transformations of each joint of Unit 3. T4‴U is the rotate angle of Unit 4. $∆d_{1}^{\prime \prime \prime }$, $∆d_{2}^{\prime \prime \prime }$, $∆d_{3}^{\prime \prime \prime }$ are the sliding transformations of each joint of Unit 4.

All this work has been done contributing to the control of nanorobotics manipulators. Since the coordinate of each Unit was derived, robot controllers will use these equations to calculate the joint values, and run the robot in order to reach the desired position.

### 3.2. Theoretial Calculation Result of the Working Space

The working space of the nanorobotics manipulator is the collection of end points in 3D space which could be reached by the end point of the end-effector. Working space is an important indicator to judge working abilities. At present, the main solutions for working out the workspace of a nanorobotics manipulator are the graphical method, the analytical method and the numerical method. Graphic visualization methods can get a cross-section of the working space or parting lines, but limited by the freedom that some three dimensional robots can't be accurately described. Analytical methods can get the spatial boundaries through multiple envelopes. Although the boundaries of the working space can be represented by the equations, the intuition is not strong, and the processing is very tedious. These methods are only applied to the situations where the number of robot joints is fewer than 3. The way that numerical methods calculate the nanorobotics manipulator’s working space is to select as many different joints of the independent variables as possible. With the forward kinematics equation, the coordinates of the endpoint values of end-effectors can be calculated, thus forming the working space of the nanorobotics manipulator. The more the number of coordinate values is, the better that the working space can be calculated to reflect the actual motion of a nanorobotics manipulator. With the development of computer software and hardware, the numerical method has been widely used since it is simple to use, and one can analyze the robot structure in any form.

Monte Carlo Method is a numerical method by means of random sampling for solving mathematical problems. This method is easier to implement with its computer graphic display capabilities, and the calculation speed is fast. This method is suitable for solving the spatial problem of an articulated manipulator, and the range of joint angles has no limit. The errors would not be influenced by the dimensions. In this paper, the working space of the manipulator was analyzed using the Matlab Monte Carlo Method.

According to the work we did above, the coordinates of each end-effector were derived. Therefore, the status and position of each AFM cantilever inside the SEM can be calculated. Based on the Monte Carlo Method, the working space of each unit can be exactly plotted. Then, we can finally get that the common working space of the end effectors is a 2.78 mm by 4.39 mm by 6 mm rectangular space. That means every point of the space can be reached by a nanorobotics manipulator. Meanwhile, collisions between manipulators may happen during the operation in this common working space. As the working space of each manipulator was calculated, these collisions could be avoided effectively during the manipulation.

## 4. Manipulation Strategy and Vision Feedback Control

### 4.1. Manipulation Strategy for CNT Handling

For nanomanipulation with the designed nanorobotics manipulation system, it is important to establish a manipulation strategy to be followed. On the one hand, the working space of cantilevers under the SEM is limited according to the calculation results in Section 3. Collisions may occur during the movement of nanomanipulators, so there is a high requirement for carefully designed path planning and sequence arrangement. On the other hand, some different challenges also arise for nanomanipulation. For large scale objects it is harder to release them than to grip them. The reason is that adhesive forces are stronger at the scale of the cantilever and samples than gravity. Therefore, handling processes, such as pick-and-place operations, need to be carefully planned. Here, we propose a manipulation strategy for picking up and placing CNTs onto other cantilevers with a designed operation sequence.

**Figure 6:**
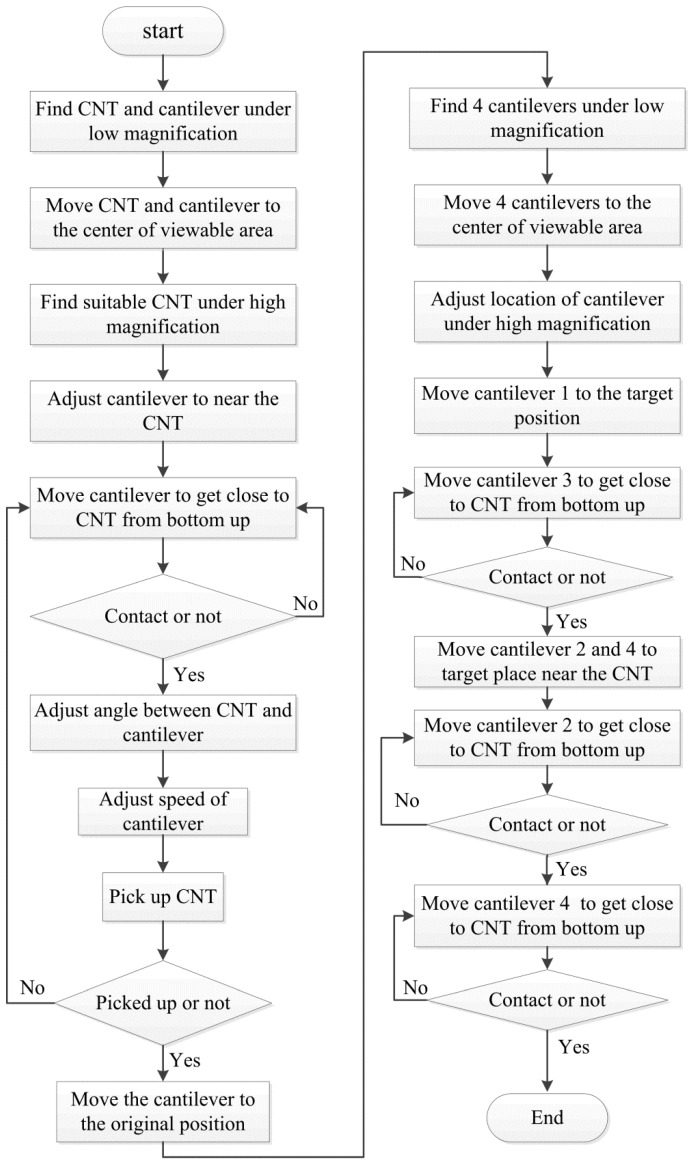
Manipulation strategy for CNT handling.

**Figure 7:**
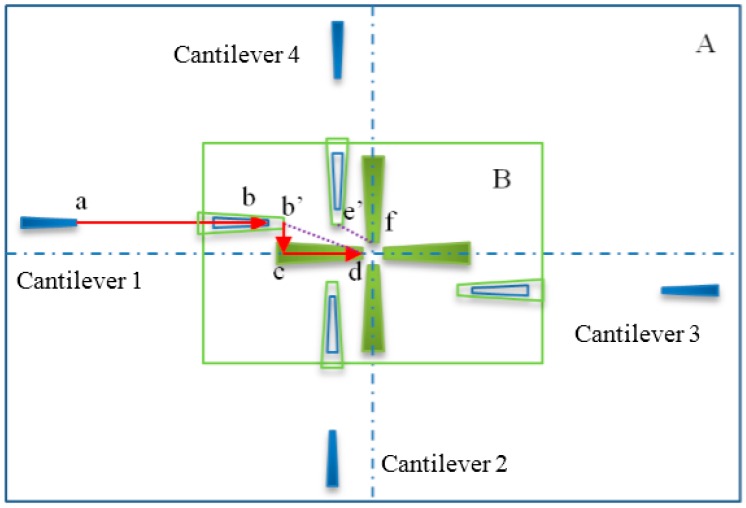
Path planning of each AFM under low and high magnification conditions. Region A refers to a visual field under low magnification. Region B refers to a visual field under high magnification. Point a is the initial position of Cantilever 1 under low magnification, point b is the target position of Cantilever 1 under low magnification, point b’and e’are the initial positions of Cantilever 1 and 4 under high magnification respectively, point c is a pocess location of Cantilever 1, piont d and f indicate the target positions of the cantilevers under high magnification.

Figure 6 depicts the manipulation strategy for CNT handling inside the SEM with the designed nanorobotics manipulation system. At the beginning, the CNT and cantilever should be found under low magnification which could allow the operation to start. Once we found the objects, we move them to the center of the viewable area for further enlargement. Then, a pure CNT with a sufficient length was selected to do the subsequent operation. After finding the suitable CNT, a cantilever was driven to the neighborhood of the CNT. Because the SEM image provides the position information of the CNT and cantilever in two dimensions, we can’t be sure whether the CNT is in contact with the cantilever or not. Hence, the cantilever was actuated to get close to the CNT from the bottom up. Once the carbon nanotube was moved with the cantilever, it indicates that these two objects are connected. Under this circumstance, a suitable pick-up angle and pick-up speed should be selected. In our previous study, the results showed that a pick-up angle at 90.1° and a pick-up speed lower than 10 nm/step would increase the probability of picking up a CNT successfully [28]. By moving the cantilever backward with a sufficient speed, CNT could be picked up effectively. When we fail to pick up the CNT, the process should be performed again back to the “bottom up” step. If a desired CNT was picked up, the cantilever would be moved to its original position. Since four cantilevers were installed inside the SEM, we should find all the cantilevers under the SEM and move them into the center of the observation area. Cantilevers must be adjusted within the depth of field of the SEM to realize a manipulation on a horizontal plane. To avoid collisions during operation, a sequence of cantilevers was arranged carefully. Cantilevers are named 1, 2, 3, 4 as shown in Figure 7 for a detailed discussion. Firstly, cantilever 1 with the CNT on it was moved to the target position according to the task. This helps to determine the location of the CNT. The opposite settled cantilever 3 was driven close to the CNT from the bottom up for the purpose of fixing it in a limited space. Finally, cantilevers 2 and 4 were moved to contact CNT, respectively.

The nanorobotics manipulators inside the SEM chamber were equipped with AFM cantilevers as end-effectors. When addressing a given task, the control system should be able to effectively plan the tasks which would be allocated to each end-effector to perform the appropriate task. In the process of task planning, the movement paths were well designed for the end-effectors to avoid collisions. The visual feedback information also has an important impact on the whole task planning process. Since the visible area of the SEM is limited, the detailed structure of the tools and objects can be observed as the magnification increased while the visible area would narrow at the same time.

**Figure 8:**
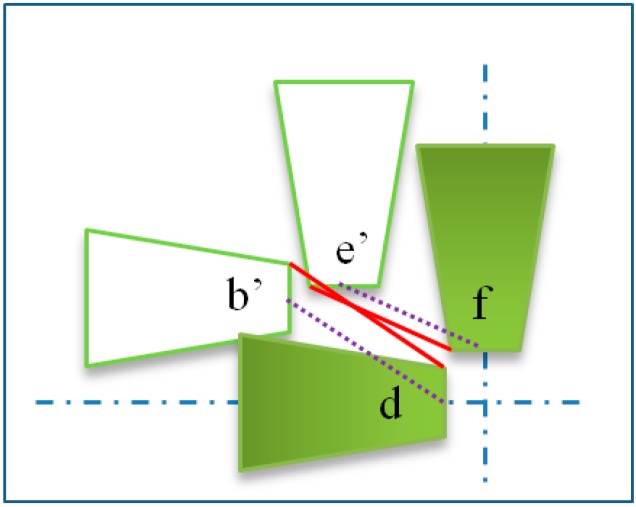
Collision diagram of AFM cantilevers. Point b’and e’are the initial position, point d and f is the target position. Delete the blank page.

Figure 7 shows the different path planning of each AFM cantilever under low and high magnification conditions. End-effectors with blue color are under low magnification presented in region A. The green colored one is under high magnification presented in region B. The center of focused beam area should remain at its position so that the center of SEM images would not change under different magnification. Actually, errors may occur during the installation of the end-effectors, as shown in region B. To perform an operation under low magnification, first of all, all end-effectors should be moved to the viewable area which is depicted in the Figure 7, where the AFM on the left in region A has moved from point a to point b. As the magnification increases, the size of the AFM that we observed from the SEM image will become larger than before. Therefore the AFM probe depicted in the Figure 7 changed its scale from b to b’ under high magnification. In the actual global coordinates, b and b′ are at the same position. Secondly, the AFM probe must be moved to the target positon d as expected under high magnification. To avoid collisions, the motion trail of AFM should be rearranged from b’ to c, then move from c to d. In addition, collisions may occur when the AFM on the left side moved directly from b′ to d and the AFM on the top moved directly from e′ to f at the same time. This situation is shown in Figure 8. The tip of the AFM is not an ideal one, it has about 6 μm in width and usually presented by the midpoint of the tip of AFM. Even though a collision doesn’t happen in AFM’s theoretical position during the movement process, it may occur during practical operation causing damage to the AFM. The red line in Figure 8 depicts the collision. Hence, such collisions must be avoided in the mission planning.

### 4.2. Visual Feedback Control inside the SEM

This section focuses on introducing the visual feedback inside an SEM since it is an important part of micro/nano manipulation. From the beginning of this decade, the SEM has been progressively proved to be a functional tool for nanomanipulation and automated nanohandling due to its high resolution, fast image acquisition time and depth of focus, so the nanomanipulation system must be equipped with the most suitable sensor. The status and position information of the CNT and AFM cantilever can be provided by SEM based on the feedback of the SEM image processing. However, it was not easy to do the image processing task. The acquisition time and quality of SEM images vary under different parameters. Taking the scan speed for example, a low scan speed can get a clearer image and the follow-up treatment is more convenient, but this has some problems in that a lot of time is wasted for saving an image which will have a negative influence on the real time processing. The image obtaioned in a high scan speed may have big noise and low reliability. Table 1 shows the scan cycles for different SEM image sizes, scan speeds and noise processing coefficients N. According to Table 3, an image size of 1024 × 768 pixels with a scan speed of 4 and a noise processing coefficient of 1 was selected.

#### 4.2.1. Automatic Binarization Method for Processing Scanned Images

Our ultimate goal is to build nanodevices with nanorobotics manipulators. Here, the status and positions of CNT and cantilevers are the key information for system control. The recognition of target objects is prerequisite. According to the morphologic features and distributions of target objects, an indirect recognition method was carried out. The cantilever would be recognized and the CNT would be chosen through clustering. End effectors which have the targets on them would be controlled to carry out subsequent manipulations after the position information is obtained.

**Figure 9:**
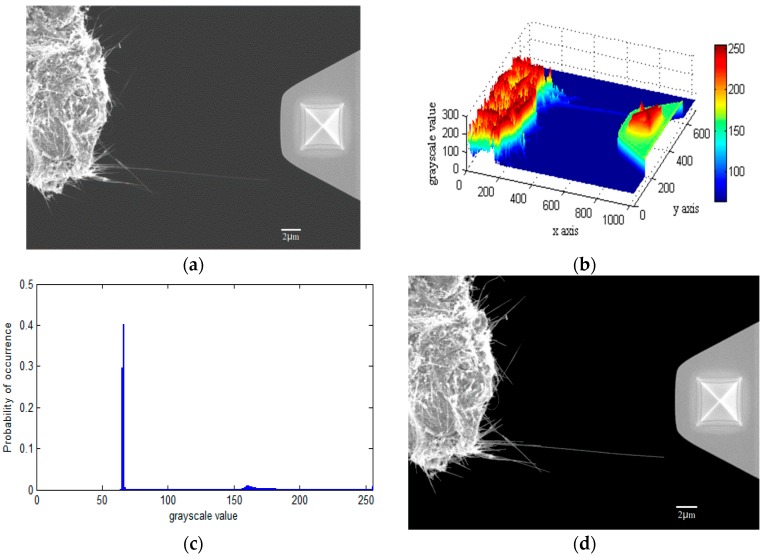
Automatic binarization for processing SEM image; (**a**) SEM images of CNTs and AFM cantilever before image processing; (**b**) Interval distribution of the pixels; (**c**) Normalized gray histogram of SEM images; (**d**) SEM images of CNTs and AFM cantilever after image processing.

The SEM images are greyscale images, as shown in Figure 9a. A distinct difference of greyscale can be observed among the CNT, the AFM cantilever and the background through pixel distribution analysis (Figure 9b). A method to separate the CNT and the AFM cantilever from the background is to set up a grey value threshold. Here, we use “thr” to represent the value of greyscale threshold, “src” to represent the SEM image pixel gray value and “dst” to represent the gray level. The formula for grayscale calculation is given below. If *src*(*x*, *y*) is larger than thr, the value of *dst*(*x*, *y*) equals to *src*(*x*, *y*), otherwise, the value of *dst*(*x*, *y*) is set as 0:

(12) $$dst(x,\;y)=\{\begin{array}{cc} src\left(x,y\right) & src\left(x,y\right)>thr\\ 0 & src(x,\;y)\leq thr \end{array}$$

However, the SEM images of different states have different brightness and contrast, and their pixel distribution also has some differences. The things above have an important influence on the information extraction of CNT and AFM in the SEM images. The automation handling process requires real-time vision feedback which means the selection of “thr” should be done automatically according to their own image pixel distribution. This process is very important for automation. Through acquisition and analysis of a large number of SEM images, their normalized gray histogram can be found as a clear peak (Figure 9c) in the low gray value area, the distribution of the high gray value area is smoother than the lower one. There is a gentle transition zone between the low gray value area and high gray value area, the threshold value “thr” is exist in the zone (Figure 9). Statistics of large number of data shows that the threshold value “thr” exists in the bottom where low gray area decreases to a gentle peak gradually. Figure 9d shows the automation threshold results.

#### 4.2.2. The Recognition of CNT and AFM Cantilever

It can be found from Figure 9 that the distribution of CNTs and AFM cantilever have a clear difference. The slender CNTs extend a long distance over the substrate. The pixels’ distribution of CNTs are sparser than those of the substrate. Given a structuring element ${\left(2k+1\right)}^{2}$, $\bar{p}$ is the pixel distribution density of the element region centered on the anchor point *p*_xy_ according to Equation (13). Where *g_ij_* is the valid value of pixel in structuring element area defined by Equation (14). The AFM cantilever is on the right side of the SEM image forming an enclosed area:

(13) $$\bar{p}=\frac{\sum_{i=x-k}^{x+k}\sum_{j=y-k}^{y+k}g_{ij}}{{\left(2k+1\right)}^{2}}$$

(14) $$g_{ij}=\{\begin{array}{ll} 1 & src\left(i,j\right)>thr\\ 0 & src(i,j)<thr \end{array}$$

**Figure 10:**
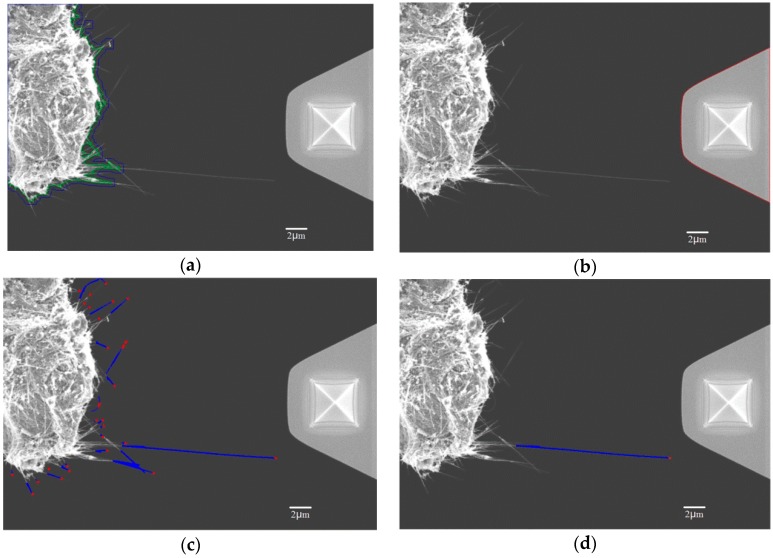
Recognition of CNT and AFM cantilever; (**a**) Schematic diagram of the new and old boundaries of the substrate; (**b**) AFM cantilever is recognized through the barycenter; (**c**) CNTs were selected from the SEM image; (**d**) The most suitable CNT was selected from the SEM image.

$p_{v}$ is the desired density of pixel distribution for distinguishing CNTs and substrate. When $\bar{p}$ is larger than $p_{v}$, the corresponding pixel belongs to the base. When $\bar{p}$ is lower than $p_{v}$, the pixel belongs to CNTs. In Figure 10, the green line is the base boundary of the substrate which can separate CNTs from the substrate. However, a CNT with a length of more than 6 μm is required according to the working space (described in Section 3) of the nanorobotics manipulators and test requirements. Moreover, it may cause interference between CNTs and AFM cantilever when the width of the end of the AFM is taken into account. CNTs haphazardly distributed in the vicinity of the substrate do not meet the requirements of the manipulation, which can be filtered by using morphological to extend the base boundary of the substrate (blue line in Figure 10a). The new boundaries of the substrate are the space limit of the AFM cantilever. Finally, the CNT can be independently segmented from the SEM image.

Among the four manipulating robots inside SEM, two opposite ones are used for the CNT pick-up process. The distribution of target objects is determined by the mechanical set up. Thus, the location of the cantilever has regularity, which is invariably displayed on the right side of the SEM image. Central moment based on contours was exerted for recognition of cantilever. *F*(*x*, *y*) is defined as a bounded and two-dimensional function, its (*p + q*) order moment is defined as:

(15) $$m_{p,q}=\int_{-\infty }^{+\infty }\int_{-\infty }^{+\infty }x^{p}y^{q}f(x,\;y)dx\;dy\;(p,q=0,\;1,\;2\ldots )$$

When *f*(*x*, *y*) is piecewise continuous in the limited area of *x − y* plane, $m_{p,q}$ sequence and *f*(*x*, *y*) would be certain mutually. (*p + q*) order central moment is defined as:

(16) $$v_{p,q}=\int_{-\infty }^{+\infty }\int_{-\infty }^{+\infty }{\left(x-x_{0}\right)}^{p}{\left(y\;y_{0}\right)}^{q}f(x,\;y)dx\;dy\;(p,q=0,\;1,\;2\ldots )$$

where ($x_{0},\;y_{0}$) is the barycenter coordinate:

(17) $$x_{0}=\frac{m_{1,0}}{m_{0,0}}$$

(18) $$y_{0}=\frac{m_{0,1}}{m_{0,0}}$$

The AFM cantilever can be recognized through the barycenter which is shown with red line in Figure 10b. Since the substrate and the AFM cantilever have been recognized from SEM images, only the CNTs remain. By using the profile feature of CNTs, they can be selected from the SEM image. As shown in Figure 10c, the blue lines represent CNTs, and the red points are the tips of the CNTs. In order to select a suitable CNT for operation it need to fulfill certain requirements. First of all, the CNT should be long enough with an effective length no less than *l*_min_. Second, CNTs should not bend too much. Thirdly, there should be no other interference around the tip of the CNT. To solve these problems, some solutions are proposed. An effective length of CNT can be selected from the tip to the branch point. The bending of CNT can be presented by the aspect ratio of the convex hull of a rectangle which surrounds the CNT. We can draw a circle with an appropriate radius centered at the tip of CNT, within which it does not contain other CNT pixels. According to these three requirements, the most suitable CNT can be selected as shown in Figure 10d.

#### 4.2.3. Closed-Loop Control of Microrobot-Based Vision Feedback

The process of controlling nanorobotics manipulators is a closed-loop control. It has the ability of self-calibration with the vision feedback system during the movement process and makes effective and accurate motion control to achieve the desired objective.

**Figure 11:**
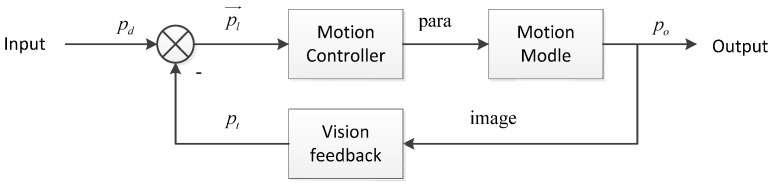
Flow chart of the closed-loop control.

**Figure 12:**
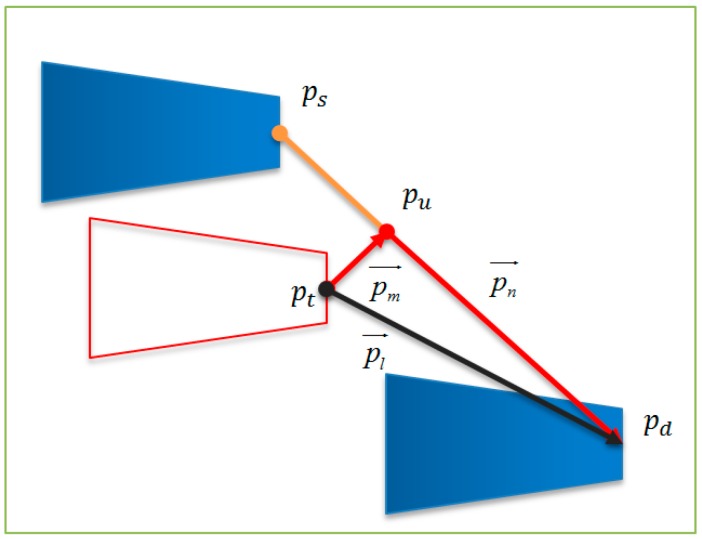
Diagram of the vectors.

In global coordinates, the position of the AFM cantilever is *p*(*x*, *y*). The process from the starting position *p_s_*(*x_s_*, *y_s_*) to the destination *p_d_*(*x_d_*, *y_d_*) is a closed-loop control, the control flow chart of which was shown in Figure 11, where *p_d_* is the expected destination, *p_o_* is the actual output location, *p_t_*(*x_t_*, *y_t_*) is the feedback position which is get by processing the SEM image. The Traject Controller will design the moving path according to the starting position and the destination. At the same time, it calculates the position error information $\vec{p_{l}}$ by the accepted feedback position. $\vec{p_{l}}$ is the error information between the planned trajectory and the feedback information *p_t_*(*x_t_*, *y_t_*), which can be represented by $\vec{p_{m}}$ and $\vec{p_{n}}$, $\vec{p_{m}}$ represents the information of AFM probe away from the planned trajectory, $\vec{p_{n}}$ indicates distance to the target position (Figure 12).

Here, $\vec{p_{m}}=p_{u}-p_{t}$, the direction of $\vec{p_{m}}$ is the deviating from the path direction, $\left|\vec{p_{m}}\right|$ is the distance of deviation. $\vec{p_{n}}=p_{d}-p_{u}$, the direction of $\vec{p_{n}}$ is the distance to the target position. Based on the error information, the Motion Controller will adjust the movement of the nanorobotics manipulators to return its planning trajectory and arrive at the specified location as expected.

## 5. Experimental Implementation

The manipulation and characterization of carbon nanotubes is of great important for building carbon nanotube-based nanodevices such as CNTFETs. As mentioned above, The nanomanipulation process is under observation in real-time with high magnification inside an SEM. Up to now, CNT picking up and placing on multi tips were performed in teleoperation mode by using the developed nanorobotics manipulation system described in Section 2. Automated control of manipulators and carbon nanotubes inside the SEM will come true by using the manipulation strategy based on the vision feedback control mentioned in Section 4.

**Figure 13:**
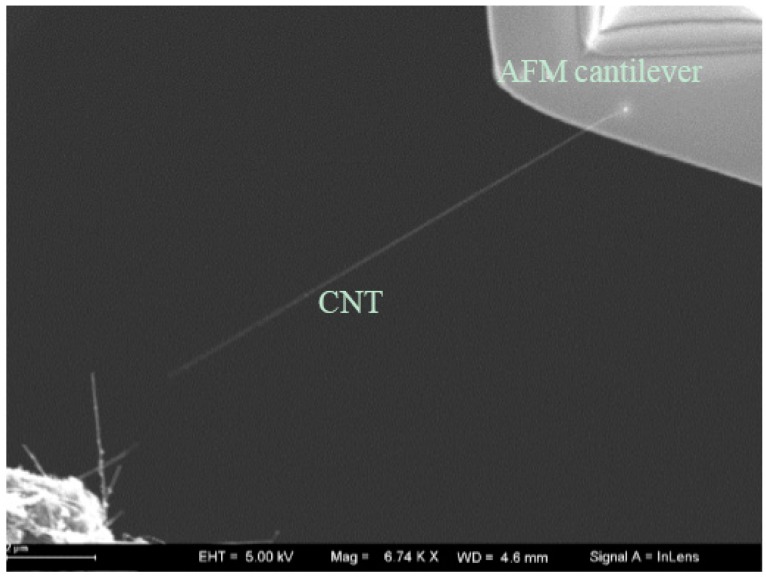
CNT picked up with AFM cantilevers.

**Figure 14:**
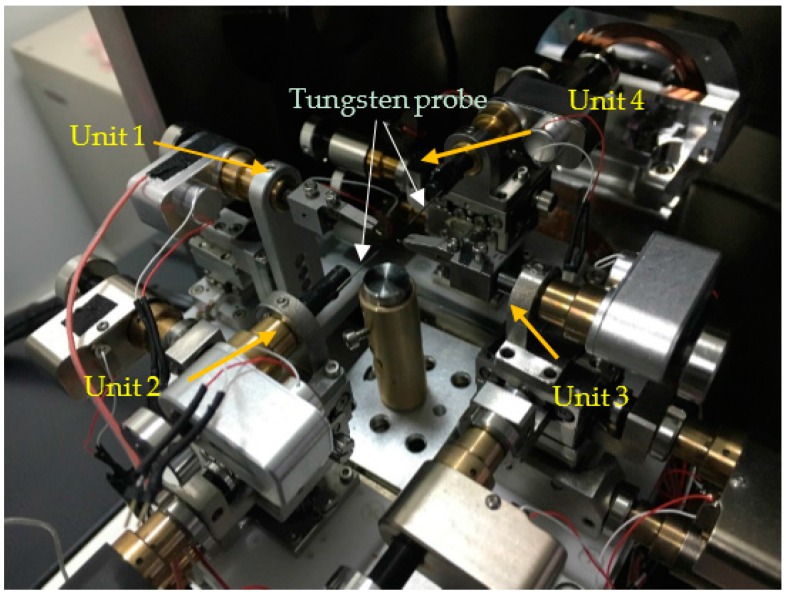
Experimental setup inside the SEM.

The application of the developed nanorobotics manipulation system implemented is the controlled pick-up and place manipulation onto multi-tip test. At first, an individual CNT picked up by the AFM cantilever in a nanorobotics manipulator is shown in Figure 13. The diameter of the CNT is between 40–60 nm. The length of the CNT is about 12 μm. Then, four nanorobotics manipulators with two AFM cantilevers and two tungsten probes as end-effectors are installed in the vacuum chamber depicted in Figure 14. End-effectors with the same probes were symmetrically arranged inside the SEM.

**Figure 15:**
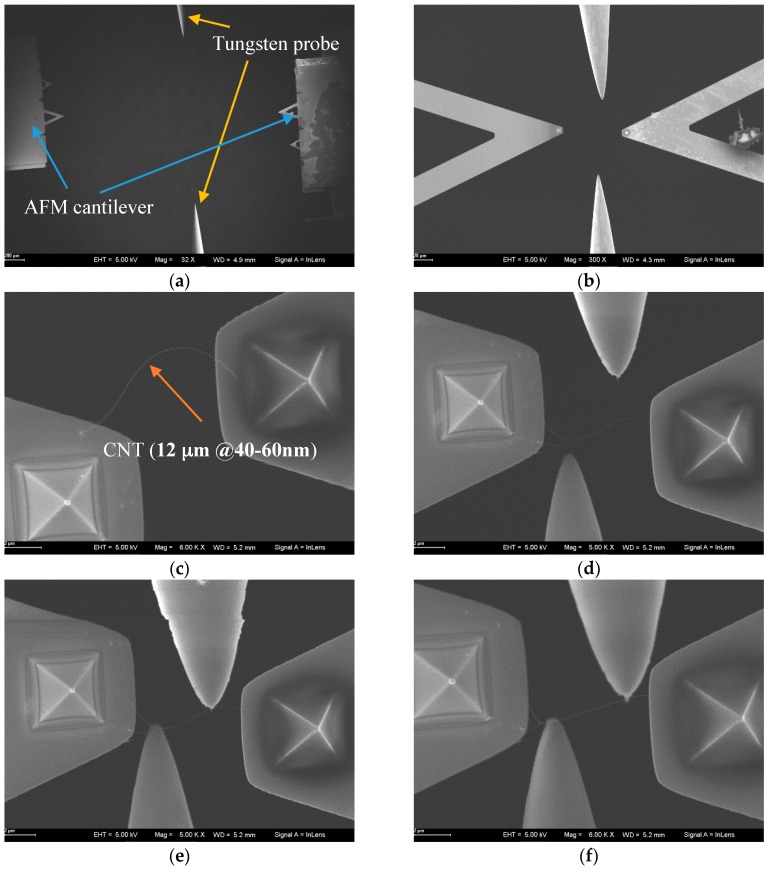
The operation process of CNT placing on four probes inside the SEM; (**a**) End-effectors of four nanorobotics manipulators are observed in SEM under low magnification; (**b**) Manipulators were controlled to drive the probe close to the center of the field with the magnification increased; (**c**) The free end of CNT is placed on the tip of the opposite AFM cantilever; (**d**) the other tungsten probes are controlled to get close to the CNT; (**e**) the probe on the top of the scene is driven to contact the CNT between two AFM cantilevers; (**f**) the probe on the bottom of the scene is driven to contact the CNT between the AFM cantilever and the tungsten probe.

CNT placement on multiple tips is carried out according to the manipulation strategy proposed in Section 4. The operation flow of the experiment is presented in Figure 15. First of all, the end-effectors of the four nanorobotics manipulators were initially adjusted to be observed in SEM under low magnification. As these four probes were moved into the visual field, the manipulators were controlled to drive the probe close to the center of the field. As the magnification increased, the four probes were controlled to reach the coarse position before manipulating the carbon nanotube. Since the working space of the manipulator is limited, collisions between probes might happen during multi-probe operation. Hence, a method to handle this problem is to control the probes one by one in a certain sequence. As a result, the opposite AFM cantilever was controlled to get close to the AFM cantilever with the CNT attached. When the free end of CNT was placed on the opposite cantilever, the rest of probes were moved to come close to the CNT. After that, the probe above the horizon was maneuvered to contact with the CNT between two AFM cantilevers. Finally, the probe on the bottom of the SEM image was driven to make fully contact with the CNT.

## 6. Conclusions and Outlook

A nanorobotics manipulation system with 16-DOF was designed and developed for 3D nanomanipulation of nanometer-scale objects inside the specimen chambers of scanning electronic microscopes (SEM). Manipulators were divided into four units. Each manipulator has 4-DOF with three linear motions in the X, Y, Z directions and a 360-degree rotational one. Manipulators were actuated with picomotors with better than 30 nm linear resolution and <1 micro-rad rotary resolution. A series of kinematics derivations of these four manipulators based on the Denavit-Hartenberg (D-H) notation were established. The common working space of the end effector was 2.78 mm by 4.39 mm by 6 mm. The manipulation strategy for picking up CNTs and placing them onto other cantilevers with a designed operation sequence was established. The vision feedback control for multi-manipulators operating inside the SEM chamber was also discussed. Finally, the application of the designed nanorobotics manipulation system was described by successfully testing of the pickup-and-place manipulation of an individual CNT onto the four probes. The experimental result have shown that a single carbon nanotube with a length of 12 μm and a diameter within 40–60 nm was successfully used in a pickup-and place task with this nanorobotics manipulation system based on the proposed manipulation strategy. In the future, the proposed control architecture will be applied to the nanorobotics manipulation system in order to automatize the sequences of the presented nanomanipulations. This will allow for a systematically prototyping of CNTFETs and other CNT-based nanodevices.

## Figures and Tables

**Table 1:** Parameters of each nanorobotics manipulator.

Parameters	Unit 1	Units 2–4
Model	SLC-1720-s/8301-UHV	TSDS-255C/8301-UHV
Dimensions (mm)	33 × 33 × 30.5/63.5 × 32.2 × 56.5	66 × 66 × 45/63.5 × 32.2 × 56.5
Travel (mm)	X ± 6, Y ± 6, Z ± 6	XY ± 3, Z ± 3
Rotate	±360º	±360º
Linear Resolution	1 nm	30 nm
Rotate Resolution	<1 micro-rad	<1 micro-rad

**Table 2:** Link parameters for Unit 1.

i	$\alpha_{i}$	$a_{i}$	$d_{i}$	$\theta_{i}$
1	−90	$a_{1}$	0	0
2	−90	0	$d_{2}$	−90
3	−90	0	$d_{3}$	180
4	0	0	$d_{4}$	−90

**Table 3:** Scan cycle under different parameters.

Image Size (Pixels)	Scanning Speed	N	Scanning Cycle
512 × 384	4	1	221.78 ms
1024 × 768	4	1	735.01 ms
1024 × 768	9	1	20.4 s
1024 × 768	4	10	7.1 s
2048 × 1536	4	1	2.7 s
